# Deep learning for religious and continent-based toxic content detection and classification

**DOI:** 10.1038/s41598-022-22523-3

**Published:** 2022-10-19

**Authors:** Ahmed Abbasi, Abdul Rehman Javed, Farkhund Iqbal, Natalia Kryvinska, Zunera Jalil

**Affiliations:** 1 Department of Creative Technologies, PAF Complex, E-9, Air University, Islamabad, Pakistan; 2 Department of Cyber Security, PAF Complex, E-9, Air University, Islamabad, Pakistan; 3 Department of Electrical and Computer Engineering, Lebanese American University, Byblos, Lebanon; 4 College of Technological Innovation, Zayed University, Abu Dhabi, United Arab Emirates; 5 Information Systems Department, Faculty of Management, Comenius University in Bratislava, Odbojárov 10, 82005 Bratislava, 25, Slovakia

**Keywords:** Computer science, Information technology

## Abstract

With time, numerous online communication platforms have emerged that allow people to express themselves, increasing the dissemination of toxic languages, such as racism, sexual harassment, and other negative behaviors that are not accepted in polite society. As a result, toxic language identification in online communication has emerged as a critical application of natural language processing. Numerous academic and industrial researchers have recently researched toxic language identification using machine learning algorithms. However, Nontoxic comments, including particular identification descriptors, such as Muslim, Jewish, White, and Black, were assigned unrealistically high toxicity ratings in several machine learning models. This research analyzes and compares modern deep learning algorithms for multilabel toxic comments classification. We explore two scenarios: the first is a multilabel classification of Religious toxic comments, and the second is a multilabel classification of race or toxic ethnicity comments with various word embeddings (GloVe, Word2vec, and FastText) without word embeddings using an ordinary embedding layer. Experiments show that the CNN model produced the best results for classifying multilabel toxic comments in both scenarios. We compared the outcomes of these modern deep learning model performances in terms of multilabel evaluation metrics.

## Introduction

Detecting possible toxicity via online communication is becoming a critical concern for social media platforms. Social media is becoming a valuable avenue for users to give their opinions, which has benefited many people, particularly minorities, by allowing them to interact and transfer knowledge and experiences^1,2^. The ability to express one’s thoughts and ideas on digital platforms is a fundamental human right that should be upheld; nevertheless, inciting and propagating toxic speech toward other groups misuses this privilege. Textual comments, including threats, insults, vulgar, insulting, offensive language, or racism, are considered toxic online discussions. Several recent research studies have been conducted on machine learning (ML) approaches to identify toxic speech in online media content^3–5^. With the rapid increase in the usage of ML algorithms for toxic comments identification, various researchers discovered that these ML classifiers are used to identify and replicate ubiquitous biases in society^6,7^.

A particular issue with many of these classification models is their sensitivity to often targeted identity groups, including Muslim, Lesbian, Jewish, gay, Black, and white, which are only harmful statements when taken with the appropriate context. A particular issue with many of these classification models is their sensitivity to often targeted identity groups, including Muslim, Lesbian, Jewish, gay, Black, and white, which are only harmful statements when taken with the appropriate context. One of the reasons for these biases in the data is an imbalanced problem in the dataset; thus, the model is over-generalized and performed classification unfairly^7–9^. We aim to enhance text classification algorithms to detect toxicity in online conversations. Using algorithms, natural language processing (NLP) extracts contextual features from a natural spoken language. The classification of text is a vital research domain in NLP. This is because textual data are widely available in our digital environment, whether in companies, hospitals, or social media platforms. Due to this, researchers are starting to conduct more studies on textual analysis tasks (topic modeling, text clustering, and classification). Nowadays, machine learning (ML) is frequently utilized for text classification. It has made significant progress, and developed novel methods such as vector embeddings, the Bag of Words model, and semisupervised and supervised ML approaches^10^. Thus, some primary issues previously faced by ML-based developed systems include handling large amounts of text data, unstructured forms of data, etc. Applying previously developed ML-based methods to billions of text documents is challenging due to the extensive computer resources and processing time required^11^.

Recently, deep learning and big data technologies are gaining popularity over time^12–14^. These innovative techniques address the constraints of previous ML approaches by using neural network models to extract meaningful information-context and important text. However, most previous works were on single-label-binary or multiclass classification issues. There has only been a little research on the multilabel categorization issue. Multilabel classification refers to the challenge of assigning the most appropriate collection of target class labels across each document from a vast number of labels, which may number in the hundreds of thousands or millions^15^.

This study provides the following contributions to identifying toxic comments effectively and efficiently by solving the constraints discussed above:

- We present deep learning (DL) methods using NLP word embeddings techniques for multilabel classification problems and produced two datasets, toxic religious comments, and race or ethnicity comments from Jigsaw Unintended Bias in Toxicity Classification.
- Evaluate the effectiveness of the proposed approach on two toxic comments datasets to train a model to predict toxic comments based on nonexclusive toxic labels and present a comparative analysis of DL models with various word embedding techniques for toxic comments classification.
- Discuss the shortcomings of deep learning algorithms for multilabel classification tasks.
- Experiments show that the CNN model with the GloVe word embeddings improves the accuracy of toxic comment classification in both scenarios compared to other DL models with different word embeddings.

“Multilable classification problem” describes the multilabel classification problem in this research work. “Literature review” summarizes past research on the issue. The description of the complete dataset is present in “Dataset selection”. The complete methodology of the proposed word is present in “Proposed approach”. “Experimental analysis and results” presents and discusses experimental results and settings. At the end conclusion and future work are presented in “Conclusion and future work”.

## Multilable classification problem

In context, the multilabel classification issue differs from the single-label multiclass classification problem. Specifically, we assign many labels to a single occurrence in multilabel classification.

This problem statement is best expressed mathematically in Eqs. (1) and (2). For instance, the function *M* depicts the mapping operation for every single document $d_{s}$ with the set of document $D^{'}$ to a specified set of target labels $T_{i}$ from the label set L:

1 $$\begin{aligned}{} & {} M : D^{'} \rightarrow T \qquad where \quad T\subseteq L = {l_{1}, l_{1},\dots , l_{k}} \end{aligned}$$

2 $$\begin{aligned}{} & {} M = {(d_{s}, T_{j})|d_{s}\varepsilon D^{'} \wedge T_{j}\varepsilon L} \end{aligned}$$

The primary distinction between single-label and multiclass categorization is that previously, only one label could be allocated to an individual instance, but subsequently, an instance may be assigned to many labels. We are working on a multilabel categorization task involving assigning document instances to several labels.

## Literature review

This section provides literature on earlier toxic text classification studies and state-of-the-art machine learning and deep learning methodologies for toxic text classification.

The presence of label correlation and the availability of many labels make the multilabel classification task more challenging. One solution is to change the task to a binary or multiclass classification problem. Various approaches are presented (copy transformation, binary relevance, pairwise comparison ranking) to convert multilabel classification to binary or multiclass classification problem^16–19^. The literature review is based on ”how different methods were used to find toxic text and other research focused on binary and multiclassification of toxic comments.” In contrast to previous methods and studies, we aimed to make a framework based on artificial intelligence and deep learning approaches for multilabel classification from religious and continental toxic comments detection.

### Monolingual toxic text detection

Previous researchers have thoroughly studied the detection of monolingual toxicity. Most research is done using English corpus^20–22^ however, Hindi, Korean, Russian, and Spanish languages are also studied^23–25^. The task can be expressed as a binary^23,26^ or multiclass^27,28^ classification problem, for example the dataset toxic comment classification challenge [https://www.kaggle.com/c/jigsaw-toxic-comment-classification-challenge] is composed of six different classes (toxic, severe-toxic, obscene, threat, insult, and identity-hate) and the dataset created for insulting/abusive language detection with three classes (sexist, neutral and racist)^27^.

### Multilingual toxic text detection

Because of the language barrier, the monolingual detection approach is inapplicable to other languages^29^. Various studies employ several ways to address the issue of the language barrier. Translating multiple languages text into single language text and extracting the semantic features from the text is one method for dealing with multilingual text^30^. The problem with this strategy is that text in several languages after translation generates noise in data and reduces data quality, which is a drawback of this technique. In this study, the author used an English language corpus to train a model, which was subsequently translated into other languages for categorization purposes^31^. The authors of this paper employ text label propagation to perform text categorization using bilingual characteristics into machine translation^32^. Compared to the classification model, which only examines monolingual texts, this strategy improves the F1 value in each class. One of the significant challenges in detecting multilingual topic text is less training data, which is more abundant in detecting monolingual toxic text^20–22^. There are two main approaches to handle this issue: transfer learning and data augmentation^33–35^.

### Machine learning methods

Although DL algorithms have grown in popularity, classical models have not vanished. Some standard ML algorithms, such as SVM, RF, NB, and LR, depend on manually derived features and cannot extract contextual data in toxic text^36–39^. In these studies, the authors suggest that LR works efficiently with low-resource settings such as less computational cost and with fewer data, whereas it is necessary to provide complete annotated data for DL classifiers^40,41^.

### Deep learning methods

Deep learning algorithms for multilabel classification have lately attained high popularity. LSTM and GRU models are two more RNN versions that are popular because they deal with the vanishing problem and reduce the gradient explosion^42,43,43^. The ability of Bi-LSTM and Bi-GRU to collect backward and forward contextual information is well established^44^. Bidirectional Encoder Representations from Transformers (BERT) is a transformer-based model. It employs a multiheaded attention mechanism. This mechanism enables the model to learn how every other word attends to a single word in a phrase to improve contextual knowledge. BERT has shown SOTA performance in a variety of NLP applications^45^, including toxic text identification^46^. One of the ways to deal with the token embeddings in a phrase is by creating a matrix to stack the phrase, which the CNN model for extracting features further processes. The embedding can occur at each level (character, word, or phrase). The authors of this study suggested a technique based on word embedding and a vector-based model named char-CNN. This technique creates character-level representations to reduce the lexicons for each language from thousands of multilingual text problems^47^.

### Model fusion

The Fusion-based technique was presented in this research^34^. This study used two distinct model representation approaches and developed two classifier techniques: improved Scrap value stream mapping (S-VSM) and the second is an interval-valued symbolic representation model. They also used unigram features with the existing study DNN model and fused two distinct models’ scores. This Fusion technique depends on pattern recognition letters and the score level.

## Dataset selection

This dataset [https://www.kaggle.com/c/jigsaw-unintended-bias-in-toxicity-classification/data] presents a multilabel classification challenge for toxic comment data from Wikipedia. It includes various difficulty levels and language diversity. The toxic comment dataset comprises around 1.8 million Wikipedia comments from Wikipedia discussion pages. Initially, the toxic comment dataset contained around 1.8 million rows and 46 columns. Apart from the target label, the dataset comprises several subtype features: severe toxic, obscene, threat, insult, identity attack, and sexually explicit. Furthermore, a subgroup of comments were categorized with the following identification characteristics to reflect the identities specified in the comment: female, transgender, male, heterosexual, another gender, lesbian or homosexual, gay, bi-sexual, other-sexual-orientation, Jewish, Christian, Hindu, Muslim, atheist, Buddhist, other religion, Asian, Black, Latino, White, other race or ethnicity, disability, physical, psychiatric or mental illness, intellectual or learning disability, other disability. Each comment in this dataset has a toxicity label between 0 and 1, signifying the percentage of human raters who believed the attribute applied to the specific comment. If the value is greater or equal to 0.5, the comment is toxic; otherwise, it is nontoxic. There are 144,334 toxic comments in the dataset, which is 8% of all the comments. The distribution of subgroup comments in the Unintended Bias in the Toxicity Classification dataset is presented in Table 1. Several studies in “Literature review” worked on subtype attributes (severe toxic, obscene, threat, insult, identity attack, and sexually explicit) for multilabel classification and obtained outstanding results. The whole dataset was not used in this study. The study focused on religious and continent base toxic comments. The dataset was split into two groups based on these two identities. Also, we write a python script that only selects rows that have at least one label. Rows that do not have labels are taken out of the dataset. Last but not least, the final data set was used for model training and prediction. The final dataset was balanced. In contrast to these standard methodologies, we developed two datasets based on identity groups using the Unintended Bias in Toxicity Classification dataset. The first is a dataset of Religiously toxic comments (RTC), while the second is a dataset of race or toxic ethnicity comments. The RTC dataset contains seven labels (Christian, Jewish, Muslim, Hindu, Buddhist, Atheist, and other religions) with 80,145 nontoxic and 11,340 toxic comments. The race or toxic ethnicity comments dataset contains five labels (Asian, Black, White, Latino, and Other race or ethnicity) with 51,555 nontoxic comments and 13,199 toxic comments. In the first step, we separate religion and race or ethnicity attributes from the original dataset, and both datasets comprise around 1.8 million rows at the start, including missing values and pending data preparation processes. After removing the missing values from the datasets, we obtain 448,000 rows, and each comment has a toxicity label between 0 and 1. Now we define a threshold: if the comment has a toxicity label value more than or equal to 0.5, it is regarded as toxic, and if it has a toxicity label value less than 0.5, it is considered nontoxic. Finally, the race or ethnicity toxic comments (RETC) dataset has 448,000 rows and six columns, whereas the RTC dataset has 448,000 rows and eight columns. In the end, the main aim of creating these two datasets is:

- To focus more on detecting toxic comments about religion, race, or ethnicity.
- To determine which religions or races/ethnicities encounter the most toxicity on the internet.
- To encourage other researchers in the area to utilize these datasets to test their methods for detecting religiously and racially poisonous remarks and multilabel categorization.

**Table 1:** Distribution of subgroup comments in unintended bias in toxicity classification dataset.

Identity group	Identity attributes	NonToxic	Toxic
Gender	Female	63,264	10,426
	Male	68,382	11,797
	Transgender	5038	1082
	Other gender	2296	427
Religion	Christian	55,915	5445
	Jewish	9290	1615
	Muslim	21,007	5643
	Hindu	1361	196
	Buddhist	1204	162
	Atheist	1974	279
	Other religion	14,710	2022
Race or Ethnicity	Asian	9746	1229
	Black	14,097	5466
	White	22,135	7813
	Latino	5,813	1123
	Other race or ethnicity	16,169	2698
Sexual orientation	Heterosexual	2735	718
	Homosexual-gay-or-lesbian	11,459	3848
	Bisexual	2800	530
	Other sexual orientation	3697	811
Disability	Physical disability	2779	448
	Intellectual or learning disability	1823	825
	Psychiatric disability or mental illness	8253	2412
	Other disability	3088	457

## Proposed approach

**Figure 1:**
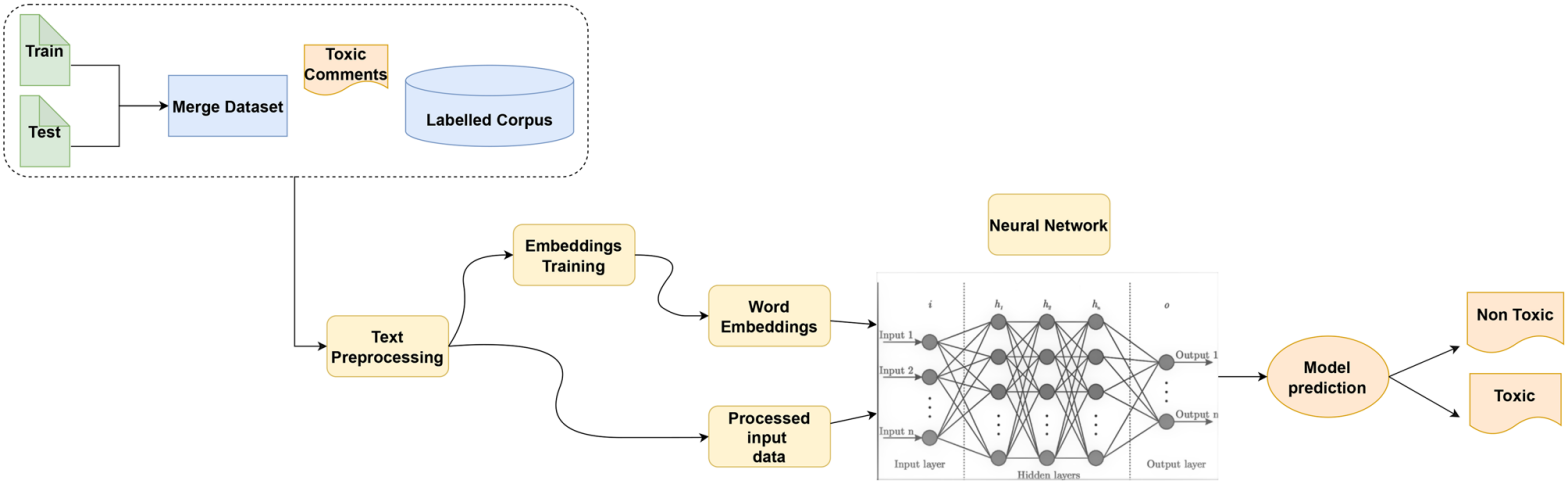
Proposed solution for toxic comments detection and classification.

The multilabel text classification (MLTC) problem and its varied applications have recently gained popularity in the last few years because of its extensive applications. Numerous machine learning approaches obtain excellent results in various classification tasks, particularly binary and multiclassification problems. Examples are video-frame identification, bioinformatics, and other applications^48^. Recently deep learning (DL) gained tremendous success in various domains. Because of their tremendous success, deep learning approaches are now commonly used in various machine learning (ML) problems. DL approaches are now employed in many machine learning applications like text classification and other applications^33^. However, compared to the standard binary or multiclass classification with multilabel classification, the multilabel classification problem is more challenging due to the correlation between labels. We employed a supervised learning technique and deep learning approaches to achieve the best results in this research. This is the most advanced approach, which is growing faster. This approach, especially the feature extraction technique, is part of natural language processing (NLP). NLP allows for achieving the most significant results. We used Keras API to implement the DL models. It used the TensorFlow platform and was implemented using Python. The approach used in this research is depicted in Fig. 1. The proposed technique consists of corpus design, data preparation and preprocessing feature extraction, and deep learning model implementation. In the first stage, the dataset is prepared to be used as input to the training model. We completed critical preprocessing procedures that will aid the DL model in understanding the data. In the next stage, a Word embedding vector is utilized that contains the text data features. This study uses Fast Text, word2vec, and global vectors for word representation (GloVe) word embeddings. In the next stage, we select the deep learning models for toxic comments classification. We utilized multiple deep learning models (NN, CNN, RNN, LSTM, and GRU) capable of excellent text classification. We used the test data to make label predictions after training the deep learning models on training data, and we acquired the label’s output after the model’s prediction.

### Data preprocessing

The original dataset contains 1,804,874 rows. Although, managing massive data presents certain issues^49^. We removed missing values from the dataset and chose just columns relating to toxic comments about religion, race, or ethnicity. We retrieved 448,000 rows after eliminating the missing data. We have some uncertainty in the dataset since some rows have zero labels, implying that all labels have a toxicity value of zero. We only choose rows with at least one toxic label to address this issue. Finally, we received 42,906 rows of hazardous religious remarks and 70,179 rows of toxic race or ethnicity comments. We discovered several duplicate rows in the dataset and removed these records. The RTC dataset contains 281 duplicate rows, while the race or ethnicity comments dataset contains 271. We then eliminated unnecessary characters and stopped words from the datasets to prevent our models from being affected.

### Word embedding techniques

Word embedding is a popular method of expressing document vocabulary. Word embedding can learn the word’s context, where words with related meanings have comparable representations. The curse of dimensionality is an essential issue that causes language modeling, and other learning tasks difficult^50^. Recently, neural network-based word embedding into a low-dimensional space was presented^51,52^. We constructed a sparse vector with a vector for each word that shows the meaning of each word. This is referred to as vector representation. In this work, we employed two ways of word embedding. It uses an embedding layer as an input layer to the model. For embeddings, the first strategy uses the Keras library. It uses an embedding layer as an input layer to the model^53,54^. This technique requires excessive time to train the model on the toxic comments dataset. The second method uses pretrained embedding techniques such as Fast Text, word2vec, and GloVe^55^. Keras provides an embedding layer for neural networks, frequently used to process text input^54^. Although each word is converted to a numeric value, the input data in the neural network model must be an integer. Keras with the tokenizer API is used for data preparation, which feeds into the model. The Google research team originally proposed word2vec. They aim to create similar aggregate models to yield word embedding. The input layer is a versatile layer that uses random weights to learn the embeddings of all words in the training data. Their approach generates vectors of each from the text corpus more efficiently than the previous method^56,57^.

The previous count base method employs statistical analysis. It finds the co-occurrence and frequency of occurrence of the word in the text corpus with its nearby words. For each word, apply these count statistics to a dense vector. Word2vec, on the other hand, used two-layer neural networks to learn word associations from a massive text corpus. It needs a massive text corpus as input. Once trained on an input text corpus, create a target vector space with hundreds of dimensions and group vectors of related words together in vector space. FastText is another pretrained word embedding technique used for text data created by Facebook’s AI Research. This model employs a shallow neural network to develop vector representations of words. The FastText allows CBOW and Skip-gram model to participate in the training process with softmax or hierarchical loss functions. By simulating character-level information for rare words, phrases, or sentences or the short sentence, it works highly effectively^58^. Stanford creates the GloVe. GloVe supports unsupervised learning. It is created to produce word embeddings by combining a corpus’s global word-word co-occurrence matrix. In this study, 100-D vectors were used for GloVe word embedding.

### Classification models and parameter settings

Deep learning has received much interest recently in the research domain because the techniques exhibit excellent results and accuracy. We evaluated the effectiveness of several DL-based classifiers in toxic comment classification tasks using the religious toxic comments and race or ethnicity comments dataset. For DL-based models, we utilized neural network (NN), convolutional neural network (CNN), recurrent neural network, long short-term memory (LSTM), and gated recurrent units (GRU)^59^. A call-back function is used during model training. This function stops the model’s training process after its performance stops increasing and monitors its performance measures for each epoch. Additionally, it gives information on the model’s internal states and numerous statistical analyses throughout training. We apply ReLU and Sigmoid two activation functions while training the model. Each neuron in the neural network has a unique weight. In a deep learning model, the activation function transforms the weighted sum of input into output shown in the fully connected layer. The ReLU activation function aids in overcoming gradient disappearance problems that are frequently linked in a deep and complex neural network during backpropagation. Therefore, it gives a more efficient method of computation while training. We may mathematically express the ReLU function R() by utilizing the max() function with the input *i*, and set of 0, defined in an Eq. (3).

3 $$\begin{aligned} {R(i) = max(0, i) } \end{aligned}$$

The sigmoid activation function is employed in this study. The sigmoid function is expressed by Eq. (4). Its representation is an ”S” shaped curve ranging from 0 to 1. The mathematical equation of the sigmoid function is defined in Eq. (4).

4 $$\begin{aligned} {\sigma (s) = \frac{1}{1+e^{(-s)}}} \qquad {for}\quad s\in R \end{aligned}$$

As the value of *s* reaches 0, the function behaves as a nonlinear with a slope. This suggests that even slight changes in predictors can significantly alter response values. The datasets presented in “Dataset selection” provide a general overview of the multilabel classification problem. In the case of the RTC dataset, it consists of 7 different labels for the *x* number of toxic comments as shown in Eq. (5):

5 $$\begin{aligned} X=\{x_{1},x_{2},....,x_{k}\} \end{aligned}$$

Moreover, Eq. (6) represents the seven various labels for RTC:

6 $$\begin{aligned} R=\{r_{1},r_{2},....,r_{k}\} \end{aligned}$$

Each religious toxic comment $X_{i}$ has allocated to labels set $R_{i}$, where $R_{i} \in \{1,2,3,4,5,6,7\}$.Experiments were carried out using the NN, CNN model, RNN, LSTM model and Gated recurrent units (GRU). We train these models on both RTC and RETC datasets, and then calculate the prediction probability $P(c_{j}| x_{i})$ of a multilabel class $c_{i}$ with respect to, $x_{i}$ as indicated in Eq. (7).

7 $$\begin{aligned} {{r^{'}_{i}} = argmax_{j \in \{1,2,3,4,5,6,7\}}P(c_{j}| x_{i})} \end{aligned}$$

#### Parameter selection

This study used multiple deep learning models, including the ReLU and sigmoid activation functions and the Adam(0.01) activation function. We employ the Adaptive Moment Estimation (Adam) activation function rather than SGD or RMSprop since it combines two gradient descent approaches. The Adam activation function computes an adaptive learning rate as an exponentially weighted average to decrease error/gradients such as momentum and an exponentially weighted average of decreasing square gradients^51^. We employed various special filters for deep learning models. We trained the model by specifying 15 epochs; if the model’s performance stops increasing, the call-back function stops training on the current epoch. The overview of special filters and dense layers is presented in Table 2.

**Table 2:** Deep learning classifiers parameter settings.

Model	Filters	Religious dataset dense layers	Race or ethnicity dataset dense layers
NN	–	50, 5	50, 7
CNN	Filters = 100 kernel size = 4	50, 5	50, 7
RNN	Units = 25	50, 5	50, 7
LSTM	Units = 25	50, 5	50, 7
Bi_LSTM	Units = 25	50, 5	50, 7
GRU	Filters = 64 Kernel size = 4 Units = 128	5	7
Bi_GRU	Filters = 64 Kernel size = 4Units = 128	5	7

## Experimental analysis and results

This study applies evaluation metrics such as accuracy, precision, recall, and F1-measure. The evaluation metrics for the single-label, binary, and multiclass classification differ from the multilabel classification. In single-label classification, simple evaluation measures are accuracy, precision, recall, and F1-score. However, in multilabel classification, a subset of predictions is given more importance and is seen as more relevant than in the absence of any prediction.

**Accuracy:** metric represents the overall expected labels correctly predicted by the classifier. This is a more balanced and superior performance metric than the hamming loss evaluation metric. Equation (8) describes the mathematical formula for this assessment metric.

8 $$\begin{aligned} Accuracy=\frac{1}{N}\sum _{i=1}^{n}\frac{|Z_{i}\cap Z_{i}^{'}|}{|Z_{i}\cup Z_{i}^{'}|} \end{aligned}$$

**Precision:** is calculated as a proportion of all positive classifications, as given in Eq. (9).

9 $$\begin{aligned} Precision=\frac{1}{N}\sum _{i=1}^{n}\frac{|Z_{i}\cap Z_{i}^{'}|}{|Z_{i}^{'}|} \end{aligned}$$

**Recall:** is also called sensitivity. It measures the proportion of correctly identified outcomes as positive when predicted, actually positive. Equation (10) presents the mathematical equation of recall.

10 $$\begin{aligned} Recall=\frac{1}{N}\sum _{i=1}^{n}\frac{|Z_{i}\cap Z_{i}^{'}|}{|Z_{i}|} \end{aligned}$$

**F1-measure:** is defined in Eq. (11). A harmonic average of recall and precision is used to calculate the F1 measure. The F1-measure considers it greatest if the value is close to or equal to one, and it is worst if the score is zero.

11 $$\begin{aligned} F_{1}-measure=\frac{1}{N}\sum _{i=1}^{n}\frac{2|Z_{i}\cap Z_{i}^{'}|}{|Z_{i}| + |Z_{i}^{'}|} \end{aligned}$$

It should be noted that higher values close to or equal to 1 correspond to greater classification quality.

### Experimental settings

we performed experiments using Kaggle, which supports Keras and TensorFlow. Furthermore, the Kaggle platform provides high-performance acceleration technologies like GPU and TPU. We performed experiments using Python-3 programming language at run-time and the GPU hardware accelerator tool, as shown in Table 3. A single classifier took approximately 2 h to train on a dataset.

**Table 3:** Computing environment.

Parameters	Values
Framework	Kaggle
Programming language	Python
GPU	NVIDIA Tesla P100 GPUs with 16GB VRAM
TPU	TPU v3-8, the core count is 8
CPU	13GB RAM + 2-core of Intel Xeon
RAM	16 GB Available
Disk	19 GB output Available

### Results and discussion

This section explains the experimental technique for evaluating performance on the RTC and RETC datasets and the benchmarked findings for general comparison. We created two datasets depending on the identity group using the Unintended Bias in Toxicity Classification dataset. Previous research in “Literature review” focused on multilabel classification subtype attributes (severe toxic, obscene, threat, insult, identity attack, and sexually explicit). This dataset aims to do multilabel classification, although there is no existing work that performs multilabel classification on religion toxic comments or race or toxic ethnicity comments. A few studies we highlight that worked using Unintended Bias in Toxicity Classification dataset subgroup comments (female, male, Jewish, Muslim, Black, White, and so on)^7,60^. Even though these studies used single-label binary classification and just one evaluation metric, the Area under the curve (AUC) score. As a result, to the best of our knowledge, no prior study has dealt with multilabel class classification utilizing RTC and RETC datasets. We used accuracy, precision, recall, and F1-score as evaluation metrics. Various deep learning models with word embedding approaches give the baseline findings. The suggested method evaluates performance in the toxic comments detection task. In this study, we used GloVe, word2vec, and FastText word embeddings to make a broader comparison with various deep learning models. In addition, we compare deep learning models that do not use the word embedding approach. Table 4 shows the results of the RTC dataset, while Table 5 shows the results of the race or ethnicity dataset.

**Figure 2:**
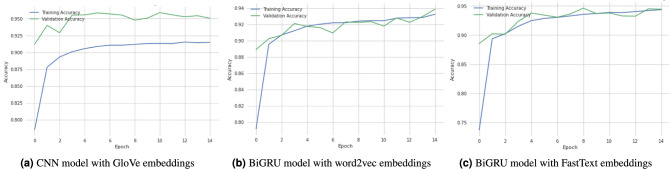
Models training and validation accuracy with word embeddings using the religious toxic comments dataset.

**Figure 3:**
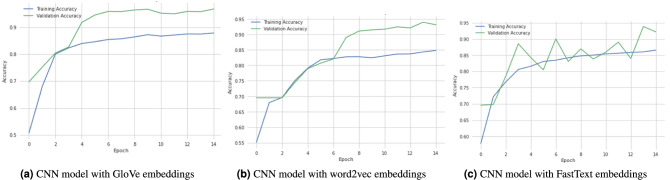
Models training and validation accuracy with word embeddings using the race or ethnicity toxic comments dataset.

#### Religious toxic comments dataset

Table 4 presents the religious toxic comments dataset results. NN CNN, RNN, LSTM, BiLSTM, GRU, and BiGRU deep learning models classify toxic religious comments. We used Glove, Word2vec, and FastText word embeddings and made a comparison of DL models with word embeddings. The CNN model with Glove word embedding has the greatest accuracy of 95.24% percent compared with the RNN, LSTM, BiLSTM, GRU, and BiGRU with glove word embeddings. Figure 2a illustrates the validation and training accuracy of the CNN model with glove embedding. As well as the precision, recall, and F1-score of the CNN model with Glove embedding are 96.59% precision, 96.91% recall, and 96.75% F1-score. While analyzing the outcomes of the word2vec technique, the BiGRU model obtains the highest accuracy of 93.64% with 97.04% precision, 94.72% recall, and 95.86% F1-score, respectively. We also plot the graph of training and validation score of the BiGRU model with word2vec as shown in Fig. 2b. Again the BiGRU model gets the best accuracy of 93.78% compared with other DL models with a FastText embedding approach. The big model with FastText embeddings obtains 97.81%, 97.06%, and 97.43% in precision, recall, and F1-score. Figure 2c shows the training and validation score of the BiGRU model with FastText embeddings. Finally, we compared several DL models that did not use word embedding techniques and discovered that the LSTM model outperformed other DL models that did not use word embedding techniques. The LSTM model’s precision, recall, and F1-score are 97.85%, 97.37%, and 97.61%, respectively, while its accuracy is 95.81%. Using the RTC dataset, we discovered that the CNN model outperforms all other DL models with embedding approaches, with an accuracy of 95.24%, the highest score compared to all other DL models with word embeddings. The LSTM model outperforms all other DL models without using any word embedding technique.

#### Race or ethnicity toxic comments dataset

The race or ethnicity toxic comments dataset consists of five multilabel classes (Asian, Black, White, Latino, and other race or ethnicity). We evaluate the performance of NN CNN, RNN, LSTM, BiLSTM, GRU, and BiGRU DL models to detect toxic comments from the race or ethnicity dataset efficiently. We used the accuracy, precision, recall, and F1-score evaluation metrics to check the classification ability of DL models.

Table 5 presents the results using the RETC dataset. Several word embeddings are used to compare deep learning algorithms for identifying toxic comments about race or ethnicity. We used Glove, Word2vec, and FastText word embeddings to compare DL models with word embeddings. According to Table 5, while using Glove word embeddings, the CNN model shows promising results with better accuracy of 96.59% and 97.49% 94.77% 96.11% precision, recall, and F1-score as compare to other DL models with Glove word embeddings. Figure 3a describes the training and validation accuracy graph of the CNN-GloVe model. Similarly, the CNN model outperforms other deep learning models using word embeddings such as word2vec and FastText. It achieves the highest accuracy of 92.91% using word2vec and 92.07% using FastText word embedding. The training and validation accuracy of CNN model with word2vec and FastText is plotted in Fig. 3b,c. In the end, the comparison of various DL models without word embeddings is also presented. The RNN model outperforms the NN, CNN, LSTM, BiLSTM, GRU, and BiGRU models. The precision-recall and F1-score of the RNN model are 96.84%, 96.72%, and 96.77%, while the accuracy of the RNN model is 94.49%. It is concluded that the CNN model performed very well in all word embedding approaches, and the RNN model outperforms other DL models without word embedding approaches to perform multilabel classification using the RETC dataset.

**Table 4:** Comparison of DL classifiers with word embeddings using religious toxic comments dataset.

Model	Accuracy%	Precision%	Recall%	F1-Score%
NN_model	94.85	98.22	95.86	96.98
CNN_model	95.04	97.63	97.52	97.57
CNN_model (GloVe)	95.24	96.59	96.91	96.75
CNN_model (word2vec)	91.61	97.86	88.82	93.11
CNN_model (FastText)	91.33	97.74	94.06	95.86
RNN_model	92.12	96.71	96.15	96.42
RNN_model (GloVe)	86.76	88.42	77.54	82.62
RNN_model (word2vec)	63.08	63.08	55.76	59.19
RNN_model (FastText)	85.58	98.14	81.73	89.17
LSTM_model	95.81	97.85	97.37	97.61
LSTM_model (GloVe)	92.46	97.21	94.16	95.65
LSTM_model (word2vec)	86.43	98.56	84.99	91.26
LSTM_model (FastText)	93.11	97.21	93.75	95.45
BiLSTM_model	92.41	97.91	97.63	97.76
BiLSTM_model (GloVe)	91.78	97.94	96.07	96.99
BiLSTM_model (word2vec)	86.43	96.02	92.16	94.06
BiLSTM_model (FastText)	92.09	97.46	95.08	96.25
GRU_model	91.90	97.47	93.46	95.42
GRU_model (GloVe)	93.13	97.53	97.37	97.45
GRU_model (word2vec)	93.16	97.80	94.42	96.08
GRU_model (FastText)	93.54	98.35	96.60	97.46
BiGRU_model	92.39	97.53	94.38	95.93
BiGRU_model(GloVe)	93.25	98.02	96.84	97.42
BiGRU_model (word2vec)	93.64	97.04	94.72	95.86
BiGRU_model (FastText)	93.78	97.81	97.06	97.43

**Table 5:** Comparison of DL classifiers with word embeddings using race or ethnicity toxic comments dataset.

Model	Accuracy%	Precision%	Recall%	F1-Score%
NN_model	86.47	97.76	96.08	96.88
CNN_model	89.45	96.80	96.78	96.78
CNN_model (GloVe)	96.59	97.49	94.77	96.11
CNN_model (word2vec)	92.91	98.91	87.76	92.99
CNN_model (FastText)	92.07	98.43	93.06	95.66
RNN_model	94.49	96.84	96.72	96.77
RNN_model (GloVe)	94.26	96.78	79.52	87.30
RNN_model (word2vec)	77.86	98.79	79.90	88.33
RNN_model (FastText)	89.70	98.86	82.33	89.82
LSTM_model	85.90	97.29	96.36	96.82
LSTM_model (GloVe)	93.64	97.88	95.33	96.58
LSTM_model (word2vec)	87.64	98.82	88.25	93.22
LSTM_model (FastText)	86.98	98.44	94.19	96.27
BiLSTM_model	83.75	97.26	96.33	96.79
BiLSTM_model (GloVe)	94.89	97.46	95.86	96.65
BiLSTM_model (word2vec)	87.64	98.86	89.71	94.05
BiLSTM_model (FastText)	87.62	98.13	95.19	96.63
GRU_model	90.76	98.77	90.19	94.27
GRU_model (GloVe)	92.87	97.78	95.99	96.87
GRU_model (word2vec)	88.89	98.30	92.92	95.52
GRU_model (FastText)	90.75	97.99	95.07	96.50
BiGRU_model	87.93	98.71	91.95	95.20
BiGRU_model (GloVe)	91.66	98.11	95.87	96.97
BiGRU_model (word2vec)	89.27	97.87	93.08	95.41
BiGRU_model (FastText)	90.26	97.45	95.70	96.56

## Conclusion and future work

In this study, we investigate several deep learning algorithms for multilabel classification. We utilized the religious and race or ethnicity toxic comments to evaluate the performance of several deep learning models. We analyzed several deep neural network architectures, including the NN, CNN, RNN, LSTM, and GRU. The study performed experiments using different DL models and compared several word embedding approaches, such as FastText, GloVe, and word2vec. The original dataset (Jigsaw Unintended Bias in Toxicity Classification) offers multilabel classification, but we discovered that earlier research mainly focused on single-label binary or multiclassification tasks, but we conducted experiments on multilabel classification tasks. Because most of the original datasets contained RTC and RETC, two datasets were constructed from the original dataset. Even though the data sets are unbalanced, the CNN model with Glove word embeddings for both datasets obtained promising results compared with other DL models and Word embedding approaches. The final results show that DL models with pretrained word embedding enhance accuracy significantly and classify toxic comments accurately. Finally, the research may be further extended by focusing on building an algorithm and strategy for dealing with imbalanced data more efficiently. As discussed above, previous research focused on single-label binary classification using subgroups (Religion, race or ethnicity, Gender, Sexual Orientation, and Disability). We want to propose an approach that classifies multilabel classification using a complete Jigsaw Unintended Bias in the Toxicity Classification dataset, including all subgroups categories. For this purpose, first, we need to extend the dataset to handle the data balancing problem. Discovering a Minority Oversampling Technique strategy in multiclass problems might be a good starting point. The method randomly selected a data point from the minority class cluster and computed the KNN neighbors for that point. The generated data points are positioned between the selected point and its neighbors. The undersampling approach may likewise be used for the predominant classes. The DNN model will give greater attention to minor class samples in this strategy. We think these two strategies will enhance the classifier’s performance by incorporating an imbalanced dataset. Finally, soft clustering approaches and strategies for tackling multilabel classification issues can be developed. The individual deep learning model takes much time for training, which is the main limitation of this study. In the future, we intend to decrease the time complexity of these models and build a framework that detects toxic comments in a short time.

## Data Availability

The datasets analyzed during the current study are available in the Kaggle repository [https://www.kaggle.com/c/jigsaw-unintended-bias-in-toxicity-classification/data].
